# Predicting tumor progression in non-functioning pituitary macroadenomas following transnasal transsphenoidal resection: insights from a single-center retrospective cohort study

**DOI:** 10.3389/fneur.2026.1763660

**Published:** 2026-04-16

**Authors:** Viktor M. Eisenkolb, Patricia Schneider, Michel Mondragon-Soto, Alexander Quiring, Bernhard Meyer, Vicki-Marie Butenschoen

**Affiliations:** 1 Department of Neurosurgery, TUM Klinikum, Technical University of Munich, Munich, Germany; 2 Department of Neurosurgery, National Institute of Neurology and Neurosurgery, Mexico City, Mexico

**Keywords:** non-functioning pituitary macroadenoma, postoperative follow-up strategy, transnasal transsphenoidal surgery, tumor progression, tumor volume

## Abstract

**Introduction:**

This retrospective, single-center study aimed to identify predictive factors for progression in postoperative residual findings of non-functioning pituitary macroadenomas (NFPMAs). The findings are intended to support individualized decisions regarding follow-up and adjuvant therapy.

**Methods:**

A retrospective analysis was conducted on 212 patients treated at a tertiary referral center between 2007 and 2023. Patients underwent MRI-guided, transnasal transsphenoidal surgery for histologically confirmed NFPMAs. Pre- and postoperative tumor volumes were assessed alongside demographic, clinical, and histopathological data. Tumor configuration was classified using Hardy and Knosp scales. Subgroups were defined based on postoperative imaging: stable residuals, progressive residuals, or recurrence after gross total resection (GTR). Statistical analyses included multivariate testing and ROC analysis to determine predictive cutoff values.

**Results:**

Of the 212 patients, initial radiological gross total resection (GTR) was achieved in 94/212 (44.3%); during follow-up, 62/212 (29.2%) had durable complete resection without recurrence, while 32/212 (15.1%) developed recurrence after initial GTR. Among patients with residual findings, 76 (64.4%) exhibited stable tumors after a mean of follow-up of 39 months, while 42 (35.6%) showed progression, which correlated significantly with larger preoperative tumor volumes (median: 11.6 cm^3^ vs. 5.81 cm^3^, *p* < 0.001). ROC analysis identified 7.12 cm^3^ as the optimal cutoff for distinguishing stable from progressive residual tumor volumes (AUC = 0.748). No significant differences between stable and progressive groups were observed in Hardy or Knosp classifications. Postoperative cortisol levels were nominally higher in patients with progressive residuals (14.10 μg/dL vs. 10.83 μg/dL; *p* = 0.022; exploratory).

**Conclusion:**

According to our study, preoperative and postoperative tumor volumes represent pragmatic prognostic markers of progression in NFPMAs, with a critical cutoff of 7.12 cm^3^ for stability. These findings could inform tailored follow-up strategies. Prospective studies are warranted to validate these results and further explore the impact of surgical and tumor characteristics on long-term outcomes.

## 1 Introduction

Pituitary adenomas are neoplasms of the adenohypophyseal cell lineage and pose the second most frequent primary brain tumors in adults (1) with a prevalence of 0.5–1/1,000 (2–5). They can be classified into micro- (<10 mm) and macroadenomas (≥10 mm). A distinction is also made between non-functioning and functioning adenomas, which describe the lack or presence of excessive production of pituitary hormones by the tumor, respectively.

Non-functioning macroadenomas represent about 30% of pituitary adenomas (6). If they are not diagnosed as incidental findings, they usually become symptomatic due to the mass effect and the symptoms caused by the compression of neighboring structures, such as visual field deficits (7) or hormonal pituitary dysfunction (8). In this case, surgical, transnasal, transsphenoidal resection is usually the gold standard treatment performed at neurosurgical centers worldwide, endoscopically or microscopically.

In addition to decompression for symptom treatment, one of the main goals is long-lasting tumor control, for which complete resection is a decisive factor. Despite developments in diagnostics and technically assisted surgical techniques in recent decades, this can only be achieved in 60% of the cases (9), depending on various factors such as invasion of the cavernous sinus (10) or the individual three-dimensional structure of the tumor.

**Figure 1:**
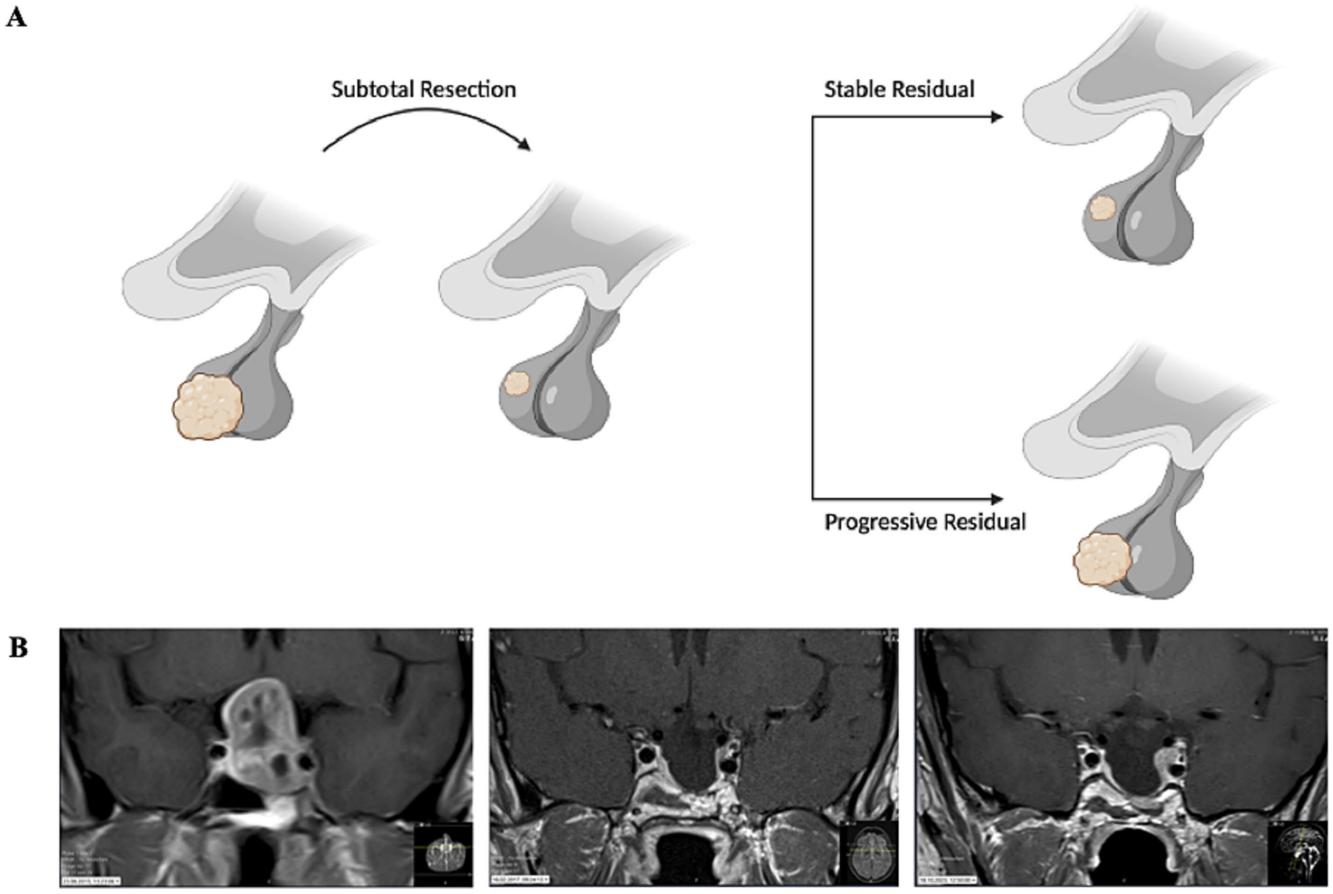
**(A)** Schematic drawing of subtotal resected pituitary macroadenomas that either remain stable during follow-up or show progression. **(B)** MRI illustrating the clinical course of a single patient over time. Coronal T1-weighted contrast-enhanced images are shown at three time points. Left: Initial scan in 2015 demonstrating a contrast-enhancing pituitary adenoma prior to the first resection. Center: Stable residual tumor adjacent to the left internal carotid artery in 2017 following subtotal resection. Right: Progressive enlargement of the known residual adenoma in 2023.

Some residual findings of the initial tumor remain stable for a long time in the follow-up imaging examinations (11). Another part, however, tends to display progressive behavior (12) (see Figure 1). To date, it is still unknown which residual tumor will show which behavior in the course of the disease. In order to take into account the very different characteristics of pituitary tumors and the significance of these differences for further therapy, the WHO changed the classification of endocrine and neuroendocrine tumors and introduced the classification of pituitary neuroendocrine tumors (PitNETs, formerly known as pituitary adenomas) in 2022 (13).

In this retrospective, single-center cohort analysis, we examine routinely collected parameters to identify factors that could predict progression in postoperative residual findings of non-functioning pituitary macroadenoma to help individualize decisions about follow-up or adjuvant therapies at an early stage of patient treatment.

## 2 Materials and methods

The results of the histopathological analyses and pre-and postoperative imaging (magnetic resonance imaging, computed tomography) were collected. Histopathological subtyping was extracted from the original pathology reports and reflects the classification terminology used at the time of diagnosis (predominantly pre-2022 WHO adenoma terminology). Proliferation markers were extracted from contemporaneous pathology reports when available. For sensitivity analyses, markers were dichotomized as follows: Ki-67 index ≥3% (yes/no), mitotic activity >2 mitoses per 10 high-power field (HPF; yes/no), and p53 positivity defined as >10 strongly positive nuclei per 10 HPF (yes/no). Because these markers were not reported in a standardized manner across the entire study period, multivariable models incorporating them were restricted to available-case subsets and interpreted as exploratory. In addition, clinical parameters such as laboratory, chemical, and endocrinological data, visual acuity, and visual field were evaluated as standard. Postoperative serum cortisol was obtained using a standardized protocol as a fasting morning blood draw at 08:00 a.m. on postoperative day 6. The institutional reference range for morning serum cortisol is 10–25 μg/dL. This is assessed in our clinic immediately pre- and postoperatively and in the first follow-up after 6–12 weeks. Extent of resection (GTR vs. STR) and postoperative baseline status were determined on the first standardized postoperative MRI performed at 6–12 weeks. This scan served as the baseline for postoperative volumetry; the timepoint was chosen to provide an early standardized baseline while reducing confounding from immediate postoperative changes compared with very early imaging. MRI was performed using a dedicated sellar protocol including contrast-enhanced T1-weighted sequences and T2-weighted imaging with thin slices. Subsequent surveillance MRI was typically performed annually. This scheme was deviated from in individual cases following interdisciplinary consultation.

Demographic factors such as sex and age were retrieved from patient files. Surgical data included microscopic vs. endoscopic approach, operation duration, and intraoperative occurrence of cerebrospinal fluid leaks. The occurrence of postoperative diabetic insipidus and revision surgery was recorded.

Tumor progression after subtotal resection (STR) was defined as clearly radiologically evident growth of residual tumor on follow-up MRI compared with the baseline postoperative MRI, as documented in the neuroradiology report and confirmed by the treating multidisciplinary team. Borderline changes were not classified as progression unless growth was confirmed on a subsequent MRI and/or deemed clinically meaningful by the multidisciplinary team. No prespecified quantitative volumetric percentage/absolute threshold was applied in this retrospective dataset. If a new tumor volume was found on follow-up imaging after gross total resection, this was declared a tumor recurrence. These cases were excluded from the primary STR progression analysis but were included in predefined sensitivity analyses.

This retrospective study included all eligible patients during the study period. In the STR cohort, progression events were limited, constraining power for small-to-moderate effects and limiting the number of predictors that can be reliably included in multivariable models (events-per-variable considerations). Therefore, we report effect sizes with 95% confidence intervals for key predictors to aid interpretation of non-significant findings (Supplementary Table S1) and explicitly acknowledge the risk of type II error.

Statistical correlation and multivariable analyses were performed using MATLAB (MATLAB version: 24.1.0.2537033 (R2024a), Natick, Massachusetts: The MathWorks Inc.; 2024). Given the number of univariable comparisons, non-volume comparisons were considered exploratory. For exploratory tests, *p*-values were additionally adjusted using the Benjamini–Hochberg false discovery rate (FDR) procedure; nominal *p*-values and *q*-values are reported (Supplementary Table S2). Missing data were handled using an available-case (complete-case per analysis) approach: patients were included in a given comparison or regression model only if all variables required for that analysis were available. No imputation was performed. Missing data proportions by variable are summarized in Supplementary Table S3, and effective sample sizes (*n*) are reported for each analysis/model. AUC confidence intervals were estimated by nonparametric bootstrapping (5,000 resamples). Positive and negative predictive values were calculated at the selected cutoff. Given heterogeneous follow-up duration, progression analyses were additionally conducted in a time-to-event framework. For STR patients, time-to-event was defined as the interval from first surgery to documented progression of residual tumor (event) or the last radiological follow-up (censoring). Progression-free survival (PFS) was estimated using Kaplan–Meier methods, and multivariable modeling was performed using Cox proportional hazards regression, reporting hazard ratios (HR) with 95% confidence intervals. Landmark PFS at 2 and 5 years was reported. In addition, an exploratory multivariable logistic regression was performed in the subset with available proliferation marker data (Ki-67, p53, mitoses; coded as defined above) to assess whether the association of preoperative volume persisted when these markers were considered.

Tumor volume and tumor extension were measured and classified by Knosp et al. for cavernous sinus invasion and by the Wilson–Hardy classification to describe tumor invasiveness through the sella floor. Pre- and postoperative tumor volumes were measured by manual slice-by-slice segmentation using iPlan Net (iPlan Net Cranial 3.0, Brainlab AG, Munich). All segmentations were performed by a single reader (blinded to outcome, if applicable), and inter-rater reliability was therefore not assessed.

## 3 Results

### 3.1 Patient characteristics

We included 212 patients out of 519 patients operated on between 2007 and 2023 with non-functioning pituitary adenomas treated via MRI-guided transsphenoidal surgery or endoscopic endonasal approach for histologically proven pituitary adenomas from 2007 to 2023 in our neurosurgical department.

**Figure 2:**
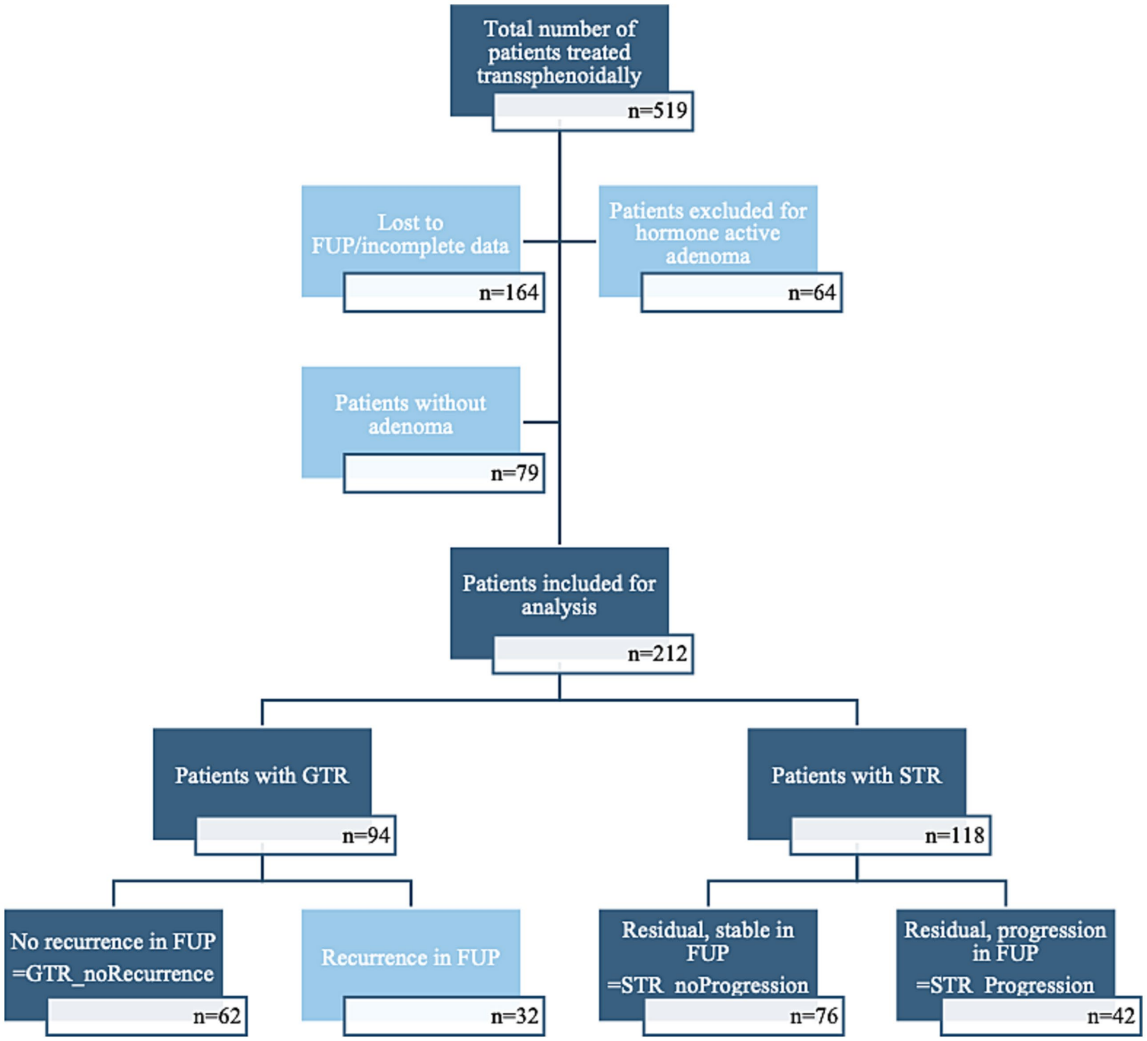
Flowchart describing the number of patients without remaining tumor, with stable remaining tumor, with progressive remaining tumor, and those excluded because of recurrence in FUP.

Of these 212 patients, 62 (29.2%) had no recurrence after gross total resection (GTR) and thus underwent only one operation during the observation period (hereafter referred to as GTR_noRecurrence, see Figure 2). In 118 patients (55.7%), only a subtotal resection (STR) of the tumor was achieved according to postoperative imaging. In a subgroup comprising 76 patients (35.8% of the total cohort, 64.4% of patients with STR), no growth of residual tumor tissue was detected by the end of 2023 (from this point onward referred to STR_noProgression). In a third cohort of 42 patients (19.8%), where only a STR of the tumor could be achieved, this remnant was progressive and led to a new resection (subsequently termed STR_Progression). Thus, progression in this subgroup reflects clinically meaningful residual growth that prompted an interdisciplinary decision for additional treatment, rather than minor volumetric fluctuations. The last group of 32 patients (15.1%) comprises patients with recurrences in whom new tumor tissue was detected in the follow-ups after the initial GTR (referred to as GTR_Recurrence). These patients were excluded from the primary STR progression analyses (see Figure 2). However, in pre-specified sensitivity analyses, we compared GTR_Recurrence to GTR_noRecurrence and performed cohort-wide models using a combined endpoint of any unfavorable outcome. In total, we therefore included 180 patients in the primary analyses.

Follow-ups were carried out a median of 2 years and a mean of 3 years after the first operation. In the STR cohort, observation time did not differ significantly between STR_noProgression and STR_Progression [median 13.0 (7–104) vs. 21.5 (7–96) months; Mann–Whitney *U* test, *p* = 0.817].

The overall mean age of our cohort was 59.17 years (25–90 years). One hundred and eleven patients (61.7%) were male, and 69 (38.2%) were female. The three subgroups did not differ significantly in age (ANOVA, *p* = 0.227) or in sex (chi-squared, *p* = 0.749) (see Table 1).

**Table 1:** Description of patient population and tumor characteristics.

Characteristic	Subgroup GTR_noRecurrence: no residual, no recurrence (*N* = 62)	Subgroup STR_noProgression: residual, stable in FUP (*N* = 76)	Subgroup STR_Progression: residual, progression in FUP (*N* = 42)	*p*-value (comparison subgroups 2 and 3)
Age (years)	Mean: 57.1 Range: 25–85	Mean: 61.3 Range: 28–83	Mean: 58.4 Range: 28–90	ANOVA, *p* = 0.275
Sex [absolute M/F (%)]	40 (64.5%)/22 (35.5%)	47 (61.8%)/29 (38.2%)	24 (57.1%)/18 (42.9%)	Chi-squared, *p* = 0.706
Hardy classification [absolute (%)]	I: 13 (21.0%) II: 30 (48.3%) III: 6 (9.7%) IV: 13 (21.0%)	I: 1 (1.3%) II: 28 (36.8%) III: 22 (29.0%) IV: 25 (32.9%)	I: 2 (5.4%) II: 14 (37.8%) III: 6 (16.2%) IV: 15 (40.6%)	Chi-squared, *p* = 0.310
Knosp classification [absolute (%)]	0: 1 (1.6%) 1: 21 (33.9%) 2: 17 (27.4%) 3: 23 (37.1%) 4: 0 (0%)	0: 0 (0%) 1: 3 (4.0%) 2: 33 (43.4%) 3: 25 (32.9%) 4: 15 (19.7%)	0: 0 (0%) 1: 2 (5.3%) 2: 13 (34.2%) 3: 12 (31.6%) 4: 11 (28.9%)	Chi-squared, *p* = 0.660
Suprasellar expansion [absolute (%)]	40 (64.5%)	67 (88.2%)	36 (85.7%)	Chi-squared, *p* = 0.926
Invasion of CS [absolute (%)]	24 (38.7%)	46 (60.5%)	19 (45.2%)	Chi-squared, *p* = 0.160
Operation method: endoscopic/microsurgical [absolute (%)]	12/50 (19.4%/80.6%)	7/69 (9.2%/90.8%)	9/33 (21.4%/78.6%)	*t*-test, *p* = 0.032
Tumor volume preoperative (cm^3^)	Mean: 5.54 Median: 4.35 Range: 0.60–37.20	Mean: 8.66 Median: 5.81 Range: 0.63–38.00	Mean: 16.98 Median: 11.60 Range: 1.87–79.90	Mann–Whitney-*U*, *p* < 0.001 Mean difference (Prog–Stable): 8.32 cm^3^ (95% bootstrap CI 3.79–13.86)
Tumor volume postoperative (cm^3^)	Mean: 0.00 Median: 0.00 Range: /	Mean: 3.45 Median: 0.95 Range: 0.10–27.90	Mean: 6.47 Median: 3.00 Range: 0.34–46.50	Mann–Whitney-*U*, *p* < 0.001 Mean difference (Prog–Stable): 2.91 cm^3^ (95% bootstrap CI 0.06–6.58)
Tumor reduction absolute (cm^3^)	Mean: 5.54 Median: 4.35	Mean: 5.25 Median: 4.27	Mean: 10.51 Median: 6.89	Mann–Whitney-*U*, *p* < 0.001
Percentage (%)	Mean: 100 Median: 100	Mean: 69.04 Median: 79.36	Mean: 65.19 Median: 65.89	Mann–Whitney-*U*, *p* = 0.365
Histopathology [absolute (%)]				
Null cell LH FSH LH + FSH ACTH Plurihormonal	32 (51.6%) 4 (6.5%) 2 (3.2%) 16 (25.7%) 4 (6.5%) 4 (6.5%)	26 (34.2%) 8 (10.5%) 6 (7.9%) 23 (30.3%) 9 (11.8%) 4 (5.3%)	21 (50.0%) 8 (19.1%) 2 (4.7%) 8 (19.1%) 3 (7.1%) 0 (0.0%)	Chi-squared, *p* = 0.178
Visual field deficit [absolute (%)]	16 (26.7%)	27 (37.0%)	19 (55.9%)	Chi-squared; *p* = 0.103
Follow-up duration (months)	Mean: 35.9 Median: 15.0 Range: 8–184	Mean: 25.1 Median: 13.0 Range: 7–104	Mean: 29.1 Median: 21.5 Range: 7–96	Mann–Whitney *U* test, *p* = 0.817

### 3.2 Surgical performance

Twenty-eight primary surgeries (15.6%) were performed endoscopically, whereas 152 patients (84.4%) were operated microsurgically. In the statistical comparison, no significant difference in approach was found between subgroup GTR_noRecurrence (proportion operated endoscopically: 19.4%) and the pooled subgroups STR_noProgression (9.2%) and STR_Progression (21.4%; *t*-test; *p* = 0.350). However, when comparing subgroup STR_noProgression and subgroup STR_Progression (9.2% vs. 21.4% endoscopic surgery), this difference was nominal (*t*-test; *p* = 0.032; Table 1) and did not remain significant after FDR adjustment (*q* = 0.080; Supplementary Table S2); it is therefore interpreted as exploratory. Interpretation is limited by the small number of endoscopic cases and likely era effects across the 16-year study period; therefore, no causal conclusions can be drawn.

Mean surgery duration was 60.3 min (20–186 min). A comparison between subgroups STR_noProgression (mean 49.6 min) and STR_Progression (mean 77.5 min) reveals a significant difference (*t*-test, *p* < 0.005).

In our center, surgery led to a GTR rate of 44.3% (*n* = 94). Intraoperative CSF flow was noticed in 64 of the 212 patients (30.2%). No statistically significant difference between subgroups was observed in this context (chi-squared, *p* = 0.403).

In eight cases, immediate postoperative surgical revisions were required: two due to postoperative hemorrhage and six due to persistent rhinoliquorrhea in the presence of intraoperative cerebrospinal fluid leakage (4.44%).

Among STR_Progression patients, 14 experienced tumor progression after re-resection during follow-up. Twelve of these patients underwent another surgery, and two opted for radiotherapy without further surgery. The follow-up procedure in this cohort was not changed from that described above.

We also analyzed the preoperative and postoperative hormone levels of subgroups STR_noProgression and STR_Progression. Postoperative cortisol values differed nominally between subgroups (*t*-test; *p* = 0.022), with higher cortisol levels in STR_Progression than in STR_noProgression; this association did not remain significant after FDR adjustment (*q* = 0.066; Supplementary Table S2) and is therefore interpreted cautiously as exploratory: subgroup STR_noProgression (mean: 10.83 μg/dL; std: 5.93 μg/dL, range: 0.7 μg/dL–25.1 μg/dL) showed lower postoperative cortisol values than subgroup STR_Progression (mean: 14.10 μg/dL; std: 5.71 μg/dL, range: 4.90 μg/dL–28.00 μg/dL).

### 3.3 Tumor configuration: Hardy and Knosp classification, suprasellar expansion and invasion of cavernous sinus

**Figure 3:**
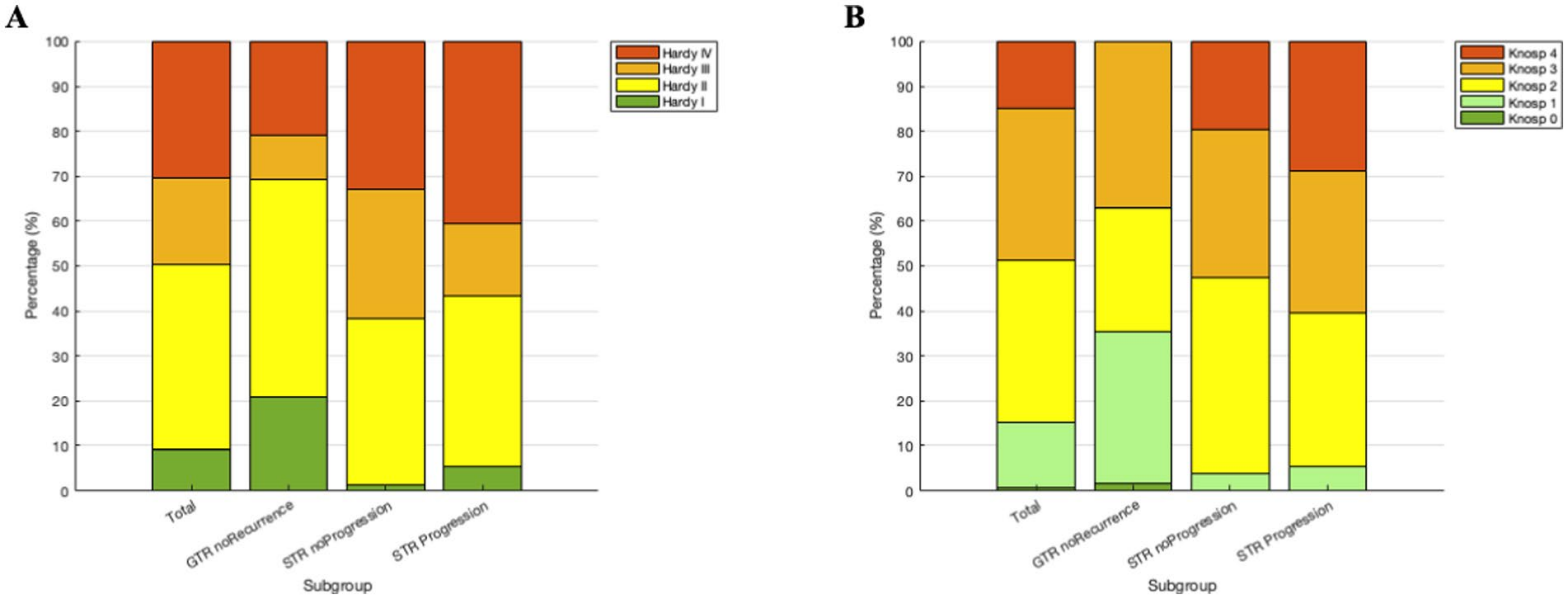
Percentage distribution of the classification according to **(A)** Hardy and **(B)** Knosp for the total cohort, the patients without residual findings, with stable residual findings and progressive residual findings.

According to the classifications of Hardy and Knosp, the percentage shares of the total cohort and the various subgroups can be seen in Figure 3.

When looking at all groups of the total cohort, there is a significant difference for both Hardy (chi-squared, *p* < 0.001) and Knosp (chi-squared, *p* < 0.001). Looking only at subgroups STR_noProgression and STR_Progression, there was no statistically significant difference between the two established classifications (chi-squared; Hardy: *p* = 0.31; Knosp: *p* = 0.66). To contextualize these non-significant findings under limited event counts, odds ratios with 95% confidence intervals are provided in Supplementary Table S1. The difference becomes significant when subgroup GTR_noRecurrence is compared with the combined subgroups STR_noProgression and STR_Progression (chi-squared; Hardy and Knosp: *p* < 0.001). When an interaction analysis is performed using chi-squared on a contingency table, it is confirmed that the Hardy classification (main effect *p* < 0.001), the Knosp classification (main effect *p* = 0.002), and also the interaction of these two classifications (*p* < 0.001) represent significant factors, contributing together to the differentiation of subgroups.

The suprasellar expansion of the tumor was also evaluated (see Table 1). There was no expansion in 37 patients (20.6%); in 143 patients (79.4%), the adenoma grew cranially beyond the borders of the sella turcica. The statistical analysis showed a significant difference between subgroup GTR_noRecurrence and the combined groups STR_noProgression and STR_Progression (chi-squared; *p* < 0.001), but no relevant difference between the latter two groups (*p* = 0.926).

When looking at the invasion of the cavernous sinus (see Table 1), there is a significant difference when comparing the total cohort (chi-squared; *p* = 0.032), but none when comparing subgroups STR_noProgression and STR_Progression (*p* = 0.160).

### 3.4 Tumor volume

Another factor that was analyzed was the tumor volume pre- and postoperatively. The preoperative and postoperative tumor volume (see Table 1) between subgroup STR_noProgression (median preoperative: 5.81 cm^3^; median postoperative: 0.95 cm^3^) and subgroup STR_Progression (median preoperative: 11.6 cm^3^; median postoperative: 3.00 cm^3^) was significantly different (Mann–Whitney *U*; preoperative and postoperative: *p* < 0.001).

**Figure 4:**
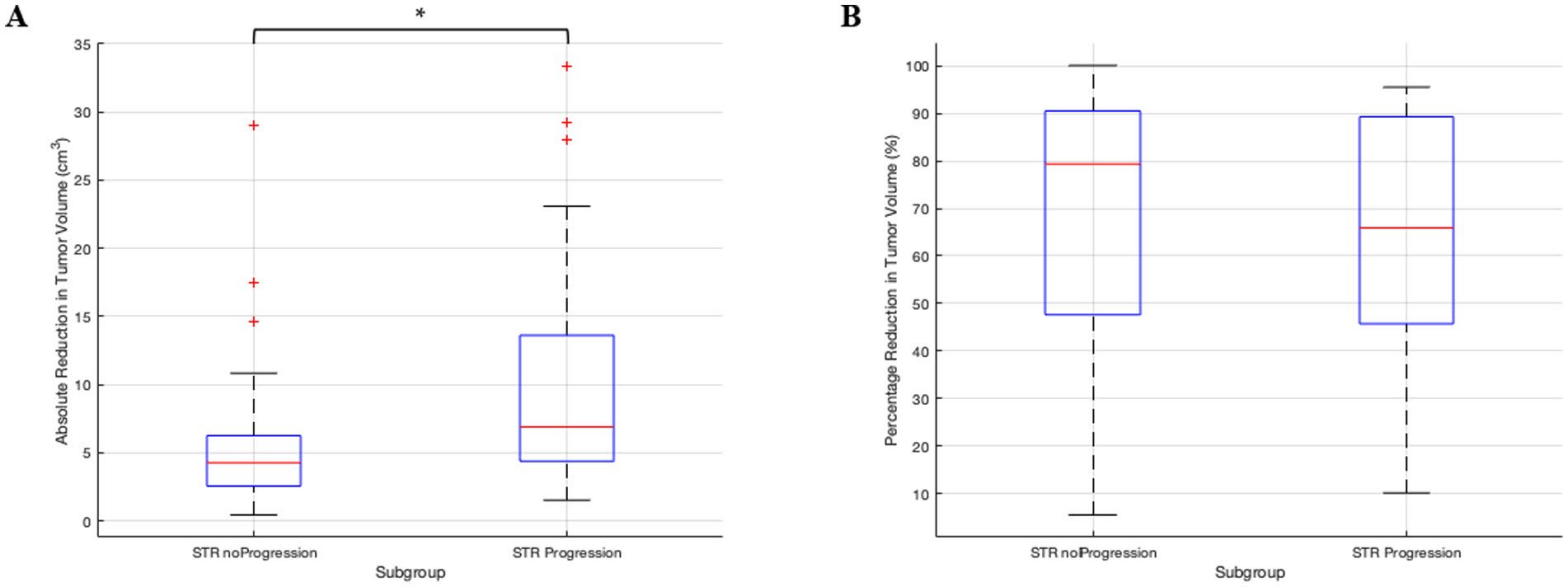
**(A)** Boxplots showing the absolute tumor reduction in cm^3^ for pre- and postoperative tumor volumes of subgroups STR_noProgression and STR_Progression. On each box, the central mark indicates the median, and the bottom and top edges of the box indicate the 25th and 75th percentiles, respectively. The whiskers extend to the most extreme data points not considered outliers. **(B)** Boxplots each show the tumor reduction percentage for subgroups STR_noProgression and STR_Progression.

The absolute difference between preoperative and postoperative tumor volume (see Figure 4A) differed significantly between these subgroups (median 4.27 cm^3^ vs. 6.89 cm^3^; Mann–Whitney *U*, *p* < 0.001). However, the percentage of tumor reduction (see Figure 4B) was only insignificantly different between subgroups STR_noProgression and STR_Progression (median 79.4% vs. 65.9%; Mann–Whitney *U*, *p* = 0.365).

**Figure 5:**
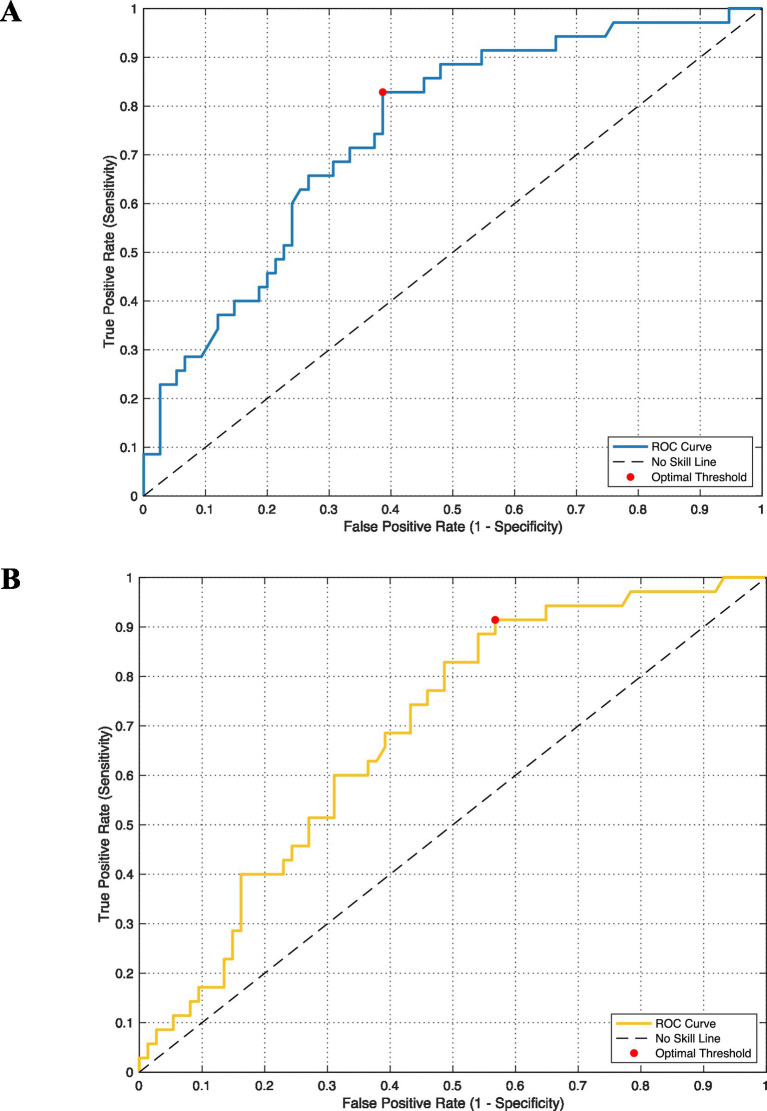
Receiver operating characteristic (ROC) curve for distinguishing between subgroup STR_noProgression and STR_Progression based on **(A)** preoperative tumor volume. The diagonal dashed line indicates a “No Skill” classifier, which predicts outcomes at random. The solid curve represents the model’s performance (AUC = 0.748; 95% bootstrap CI 0.649–0.840). A red dot marks the optimal threshold, calculated using the Youden index, which balances sensitivity and specificity. **(B)** Same as in **(A)** but based on postoperative tumor volume (AUC = 0.689; 95% bootstrap CI 0.585–0.786); the Youden-optimal cutoff was 0.68 cm^3^ (red dot).

To calculate the association between preoperative tumor volume and the likelihood of postoperative classification into groups STR_noProgression and STR_Progression, we performed a receiver operating characteristic (ROC) analysis (see Figure 5A) using cases with available volumetry (*n* = 110). The ROC analysis yielded an area under the curve (AUC) of 0.748 (95% bootstrap CI 0.649–0.840). Based on the ROC analysis and the Youden index, the optimal cutoff value for preoperative tumor volume to distinguish between the subgroups STR_noProgression and STR_Progression was determined to be 7.12 cm^3^. At this threshold, the sensitivity was 82.9% and the specificity was 61.3%. At the same cutoff, the positive predictive value was 50.0% and the negative predictive value was 88.5% (progression prevalence 31.8% in this subset). Patients with a tumor volume ≤7.12 cm^3^ were more likely to be classified into subgroup STR_noProgression, whereas those with a tumor volume >7.12 cm^3^ were more likely to be classified into subgroup STR_Progression. ROC analyses are reported as descriptive risk stratification metrics in addition to the primary time-to-event analyses.

Using the same approach for postoperative residual tumor volume (Figure 5B), the ROC analysis identified an optimal cutoff of 0.68 cm^3^, yielding an AUC of 0.689 (95% bootstrap CI 0.585–0.786). Patients with a residual volume ≤0.68 cm^3^ were more likely to be classified as STR_noProgression, whereas those with >0.68 cm^3^ tended to be classified as Group STR_Progression; at this threshold, sensitivity was 91.4% and specificity 43.2%.

As a time-aware sensitivity analysis, landmark ROC analyses yielded an AUC of 0.809 at 24 months (95% bootstrap CI 0.647–0.942; *n* = 48) and 0.646 at 60 months (95% bootstrap CI 0.439–0.837; *n* = 34), excluding censored patients with follow-up shorter than the landmark.

To assess independent associations with progression, we performed forced-entry multivariable models in the STR cohort using available-case data (Supplementary Table S4). In logistic regression, preoperative tumor volume remained significantly associated with progression after adjustment for surgical approach and postoperative residual volume, whereas surgical approach did not remain an independent predictor. In Cox time-to-event modeling, the association of preoperative volume was attenuated after adjustment for postoperative residual volume.

### 3.5 Sensitivity analysis including recurrence after apparent GTR

Preoperative tumor volume was higher in patients with recurrence after apparent GTR (GTR_Recurrence) compared to GTR_noRecurrence (median 7.93 vs. 4.35 cm^3^; Mann–Whitney *U*, *p* = 0.0218; cases with available volumetry). In a cohort-wide analysis using “any unfavorable outcome” (GTR_Recurrence or STR_Progression) as endpoint, preoperative volume showed discriminative ability (ROC AUC 0.716; bootstrap 95% CI 0.630–0.799; Youden-optimal cutoff 9.18 cm^3^). In multivariable logistic regression adjusting for postoperative residual volume, preoperative volume remained significant within the STR cohort and in cohort-wide models (see Supplementary Tables S5, S6).

Proliferation markers were available only in a subset of patients and were not standardized across the full study period (Ki-67 ≥3%, mitoses >2/10 HPF, p53 positivity >10 strongly positive nuclei/10 HPF). In the STR cohort subset with available Ki-67 and p53 (*n* = 39; events = 11), multivariable logistic regression showed that preoperative tumor volume remained associated with progression (OR 1.148 per 1 cm^3^, 95% CI 1.016–1.298; *p* = 0.0269), whereas Ki-67 and p53 did not reach statistical significance and had wide confidence intervals (Supplementary Table S7). In a whole-cohort subset with available Ki-67, p53 and mitoses (*n* = 59; events = 16) using “any unfavorable outcome” (GTR_Recurrence or STR_Progression) as endpoint, preoperative volume remained associated with outcome (OR 1.181 per 1 cm^3^, 95% CI 1.057–1.318; *p* = 0.00316), while proliferation markers were imprecisely estimated (Supplementary Table S7). These results are considered exploratory due to limited subset size.

### 3.6 Time-to-event analysis

Kaplan–Meier analysis was used to estimate progression-free survival (PFS) after subtotal resection (STR), censoring patients without progression at last radiological follow-up (Supplementary Figure 1). Landmark PFS estimates were 0.762 at 24 months and 0.566 at 60 months, and the median PFS was 73 months. Cox proportional hazards regression was used for multivariable modeling (hazard ratios with 95% confidence intervals).

### 3.7 Histological subtype and preoperative visual field deficit

Postoperative histopathologic analysis and subtype classification were also assessed (see Table 1). There was no statistically relevant difference between patients in subgroups STR_noProgression and STR_Progression (chi-squared: *p* = 0.178; multinomial logistic regression: *p* = 0.111).

If we compare the numbers of the preoperative visual field defects for subgroups STR_noProgression and STR_Progression (see Table 1), they only differ insignificantly from each other (chi-squared; *p* = 0.103). For interpretability despite limited event counts, effect estimates (odds ratios) with 95% confidence intervals for these comparisons are reported in Supplementary Table S1.

## 4 Discussion

Our data indicate that preoperative absolute tumor volume predicts progression of residual disease in non-functioning pituitary macroadenomas. The ROC analysis yielded an AUC of 0.748 and a cutoff of 7.12 cm^3^ (Figure 5A). Patients who later showed progressive residuals already had larger tumors preoperatively (median 11.60 cm^3^ vs. 5.81 cm^3^). A methodological consideration is the radiographic distinction between GTR and very small residual disease on early postoperative imaging. Despite using the first standardized follow-up MRI at 6–12 weeks as baseline, small residuals may remain occult and later manifest as “recurrence after apparent GTR.” To address this, we performed sensitivity analyses including these cases, which supported preoperative tumor volume as a prognostic marker across the entire cohort. Future prospective studies could incorporate delayed baseline imaging (e.g., 3–6 months) to further reduce uncertainty from postoperative changes and improve classification of small residuals.

Although the percentage reduction was similar between groups (median 79.4% vs. 65.9%; *p* = 0.365; Figure 4B), the absolute residual was greater in those with progression (median 3.00 cm^3^ vs. 0.95 cm^3^; *p* < 0.001; Table 1). Taken together, the absolute postoperative residual volume appears to be more informative for predicting progression than the percentage of reduction.

Postoperative residual volume showed modest discriminative ability (AUC 0.689) with a Youden-optimal cutoff of 0.68 cm^3^ (Figure 5B). This identifies most progressive cases but with limited specificity, supporting its use as one component of postoperative risk stratification. Because all patients with residual NFPMA require imaging surveillance, the proposed volume cutoff is not intended to indicate immediate adjuvant therapy. Rather, it may support risk-adapted follow-up intensity (e.g., shorter early imaging intervals and earlier multidisciplinary review in patients above the cutoff). This is in line with current literature (14–16). Nevertheless, we found no association between suprasellar extension and post-STR behavior (14). Further analyses could be carried out here to objectify the extent of suprasellar growth.

When evaluating the pre- and postoperative hormone values, only the postoperative cortisol value stood out. Here, a higher value was found in subgroup STR_Progression. Because multiple exploratory comparisons were performed, this cortisol association should be interpreted cautiously and considered hypothesis-generating.

According to our data, both the Hardy and Knosp classifications, and invasion of the cavernous sinus can only be used to estimate the likelihood of partial resection of non-functioning pituitary macroadenomas. These parameters fail to reach statistical significance when comparing the cohort with progressive residual findings (STR_Progression) to those with stable residual findings (STR_noProgression).

There are various opinions in the literature regarding the superiority of endoscopy compared to microsurgical transnasal transsphenoidal resection (17–19). In our cohort, endoscopic resection was associated with a higher progression rate over time compared to microsurgical transnasal transsphenoidal resection, while the rate of complete resections remained similar. In multivariable models in the STR cohort (Supplementary Table S4), surgical approach (endoscopic vs. microsurgical) did not remain an independent predictor after adjustment for tumor volume metrics. Given the markedly unbalanced distribution, the small number of endoscopic cases and potential era effects over the study period, this finding should be interpreted cautiously.

The histological subtype and its role in the progression of pituitary adenomas remains a topic of ongoing debate. In our cohort, no statistically significant relevance could be confirmed in this regard, which aligns with the findings of some studies (20–22) while contradicting others (23–25), for example, stating that plurihormonal adenomas and silent ACTH or GH/PRL adenomas relapse more frequently. Histopathological categories were taken from contemporaneous reports and were not systematically reclassified according to WHO 2022 PitNET criteria; future studies with standardized immunohistochemical profiling and access to original material may refine subtype–outcome associations. With new advancements in histological processing and molecular analyses, our cohort could be further examined.

The mean age in our cohort was 59 years. This aligns with relevant literature (26, 27) and previous studies conducted at our center (7, 28). The slight overrepresentation of men (61.7%) is also consistent with other studies (26). There are no statistically significant differences between the subgroups concerning these parameters (see Table 1).

Another clinical factor we analyzed was the presence of a preoperative visual field defect. We could not identify a significant difference between subgroups STR_noProgression and STR_Progression. Thus, based on our cohort, no conclusions can be drawn regarding the further course of postoperative residual findings.

Additional limitations include the retrospective nature of the analysis, the single-center cohort, the varying lengths of follow-up periods, and the naturally smaller number of patients with progressive residual findings. Conventional ROC analyses treat progression as binary and do not fully account for censoring; therefore, we emphasize time-to-event analyses as primary and report ROC (including landmark ROC) as secondary descriptive measures. Given heterogeneous follow-up duration, we complemented binary progression analyses with time-to-event methods (Kaplan–Meier and Cox regression) that explicitly account for censoring. A further limitation is that, in this retrospective cohort, no prespecified quantitative growth threshold (e.g., ≥20% volume increase) was applied. Although borderline changes required confirmation on subsequent imaging and progression in subgroup STR_Progression was treatment-triggering, subtle changes close to the measurement error could theoretically have been misclassified. Prospective studies using predefined volumetric criteria are warranted. Because volumetry was based on manual segmentation by a single reader, inter-rater reliability could not be assessed and reader-dependent measurement variability cannot be excluded. A further limitation is missing data for selected variables, which reduces precision and may introduce bias; results are therefore interpreted cautiously. Given multiple exploratory comparisons, nominal *p*-values for secondary variables are interpreted cautiously. In addition, the Ki-67 proliferation index was not available in a standardized manner for most patients, and related proliferation parameters (p53, mitotic activity) were also inconsistently reported. Therefore, these markers could not be incorporated comprehensively into our multivariable models, and Ki-67 represents a potential unmeasured confounder. Accordingly, we interpret tumor volume as a pragmatic imaging-based risk marker that likely captures resectability and residual burden; prospective studies with standardized Trouillas-based profiling are needed to evaluate independence from proliferation markers.

We demonstrate in our large cohort that both preoperative and postoperative tumor volume play a role in predicting whether a residual tumor after surgery will remain stable or progress over time. In the meantime, 65% of the patients with STR remained without tumor progression. According to our data, various imaging identifiable factors, such as the Hardy and Knosp classification, whether the tumor extends suprasellar or invades the cavernous sinus, can only be used to estimate the likelihood of complete resection. Further studies are needed to analyze the role of histological subtypes.

## 5 Conclusion

In estimating the risk of a progressive residual finding following transnasal transsphenoidal resection of a non-functioning pituitary macroadenoma, our findings support preoperative and postoperative tumor volume as pragmatic imaging-based prognostic markers and potential parameters for risk-adapted follow-up strategies. After further analysis, these findings could be integrated into individualized decision-making for subsequent diagnostic and therapeutic strategies, serving as one component of a risk-adapted follow-up concept.

## Data Availability

The raw data supporting the conclusions of this article will be made available by the authors, without undue reservation.
