# Supplementary material for: Predicting tumor progression in non-functioning pituitary macroadenomas following transnasal transsphenoidal resection: insights from a single-center retrospective cohort study
Source: Front Neurol. 2026 Apr 16;17:1763660. doi: 10.3389/fneur.2026.1763660 (PMC13128430; doi:10.3389/fneur.2026.1763660)
Supplement: Supplementary file 1 [file Supplementary_file_1.DOCX]

Supplementary Material

# Supplementary Data

Supplementary Material should be uploaded separately on submission. Please include any supplementary data, figures and/or tables.

Supplementary material is not typeset so please ensure that all information is clearly presented, the appropriate caption is included in the file and not in the manuscript, and that the style conforms to the rest of the article.

# Supplementary Figures and Tables

2.1 Supplementary Tables

**Supplementary Table S1.** Effect sizes for candidate predictors with non-significant univariable comparisons in the STR cohort. Odds ratios (OR) with 95% confidence intervals quantify the range of effects compatible with the data and facilitate interpretation of non-significant results under limited event counts. Binary predictors were analyzed using Fisher’s exact test; when a zero cell occurred, a 0.5 continuity correction was applied for OR/CI estimation. Histology was tested using a chi-square test (6×2 table); category-specific contrasts are provided descriptively due to sparse cells. Analyses used available-case data due to missingness.

|  | **Subgroup STR_noProgression** | **Subgroup STR_Progression** | **p-value** |
| --- | --- | --- | --- |
| **Hardy grade (binary)** (≥3 vs <3) | ≥3: 47/76 (61.8%)  <3: 29/76 (38.2%)  n=76 | ≥3: 21/37 (56.8%)  <3: 16/37 (43.2%)  n=37 | p=0.684  nOR 0.810 (95% CI 0.365–1.799) |
| **Knosp grade (binary)** (≥3 vs <3) | ≥3: 40/76 (52.6%)  <3: 36/76 (47.4%)  n=76 | ≥3: 23/38 (60.5%)  <3: 15/38 (39.5%)  n=38 | p=0.549  nOR 1.380 (95% CI 0.626–3.044) |
| **Visual field deficit (pre-op)** (GF_pre = 2 vs 0; 0 = no deficit) | Deficit: 27/73 (37.0%)  no Deficit: 46/73 (63.0%)  n=73 | Deficit: 19/34 (55.9%)  no Deficit: 15/34 (44.1%)  n=34 | p=0.093  nOR 2.158 (95% CI 0.944–4.935) |
| **Histology (overall distribution)** (6 categories) | see Table 1 | see Table 1 | p=0.178 (χ², df=5) |
| **Histology: LH vs Null cell** (ref = category Null cell) | 8 vs 26 \nn=76 | 8 vs 21 \nn=42 | p=0.777 \nOR 1.238 (95% CI 0.397–3.857) |
| **Histology: category FSH vs Null cell** (ref = category 0) | 6 vs 26 \nn=76 | 2 vs 21 \nn=42 | p=0.446 \nOR 0.413 (95% CI 0.075–2.260) |
| **Histology: category LH + FSH vs Null cell** (ref = category 0) | 23 vs 26 \nn=76 | 8 vs 21 \nn=42 | p=0.102 \nOR 0.431 (95% CI 0.160–1.158) |
| **Histology: category ACTH vs Null cell** (ref = category 0) | 9 vs 26 \nn=76 | 3 vs 21 \nn=42 | p=0.326 \nOR 0.413 (95% CI 0.099–1.720) |
| **Histology: category plurihormonal vs Null cell** (ref = category 0; 0.5 correction applied) | 4 vs 26 \nn=76 | 0 vs 21 \nn=42 | p=0.134 \nOR 0.137 (95% CI 0.007–2.687) |

**Supplementary Table S2. Multiple-testing adjustment (Benjamini–Hochberg false discovery rate, BH-FDR) for exploratory univariable comparisons in the STR cohort (STR_noProgression vs STR_Progression).** *Nominal p-values are taken from Table 1 / text. BH-FDR q-values were computed across the exploratory tests listed below. For results reported as p<0.001 and p<0.005, conservative values of p=0.001 and p=0.005 were used for q-value calculation.*

|  | **Nominal p-value** | **BH-FDR q-value** |
| --- | --- | --- |
| **Tumor volume preop [cm³]** | <0.001 | 0.0050 |
| **Postoperative residual volume [cm³]** | <0.001 | 0.0050 |
| **Tumor reduction (absolute)** | <0.001 | 0.0050 |
| **Operation duration** | <0.005 | 0.0188 |
| **Postoperative cortisol** | 0.022 | 0.0660 |
| **Operation method (endoscopic vs microsurgical)** | 0.032 | 0.0800 |
| **Visual field deficit (GF_pre)** | 0.103 | 0.2207 |
| **Cavernous sinus invasion** | 0.160 | 0.2967 |
| **Histology (subtype; overall)** | 0.178 | 0.2967 |
| **Age** | 0.275 | 0.4125 |
| **Hardy classification** | 0.310 | 0.4227 |
| **Tumor reduction (percentage)** | 0.365 | 0.4563 |
| **Knosp classification** | 0.660 | 0.7564 |
| **Sex** | 0.706 | 0.7564 |
| **Suprasellar expansion** | 0.926 | 0.9260 |

**Supplementary Table S3.** Missing data by variable and subgroup. Values are shown as missing/total (%).

|  | **Subgroup GTR_noRecurrence N=62** | **Subgroup STR_noProgression N=76** | **Subgroup STR_Progression N=42** |
| --- | --- | --- | --- |
| **Age** | 0/62 (0.0 %) | 0/76 (0.0 %) | 0/42 (0.0 %) |
| **Sex** | 0/62 (0.0 %) | 0/76 (0.0 %) | 0/42 (0.0 %) |
| **Hardy Classification** | 0/62 (0.0 %) | 0/76 (0.0 %) | 5/42 (11. 9%) |
| **Knosp Classification** | 0/62 (0.0 %) | 0/76 (0.0 %) | 4/42 (9.5 %) |
| **Histopathology** | 0/62 (0.0 %) | 0/76 (0.0 %) | 0/42 (0.0 %) |
| **Visual field deficit** | 2/62 (3.2 %) | 3/76 (3.9 %) | 8/42 (19.0 %) |
| **Operation Method** | 0/62 (0.0 %) | 0/76 (0.0 %) | 6/42 (14.3 %) |
| **Tumor volume preop** | 3/62 (4.8 %) | 1/76 (1.3 %) | 7/42 (16.7 %) |
| **Tumor Volume postop** | 0/62 (0.0 %) | 2/76 (2.6 %) | 7/42 (16.7 %) |
| **Follow up Duration** | 8/62 (12.9 %) | 14/76 (18. 4%) | 14/42 (33.3 %) |
| **Operation duration** | 0/62 (0.0 %) | 0/76 (0.0 %) | 6/42 (14.3 %) |
| **Intraoperative CSF leak** | 0/62 (0.0 %) | 0/76 (0.0 %) | 6/42 (14.3 %) |
| **Suprasellar Expansion** | 0/62 (0.0 %) | 0/76 (0.0 %) | 0/42 (0.0 %) |
| **Invasion of CS** | 0/62 (0.0 %) | 0/76 (0.0 %) | 0/42 (0.0 %) |

**Supplementary Table S4.** Multivariable models for progression after STR (STR_noProgression vs STR_Progression). Analyses were restricted to the STR cohort. Covariates were entered using a forced-entry strategy based on a priori clinical relevance (preoperative tumor volume, postoperative residual volume, surgical approach). Continuous predictors are reported per 1 cm³ increase. Available-case analysis was used (patients with missing covariates were excluded from the corresponding model). Pre- and postoperative volume measures were strongly correlated (r=0.834); therefore, interpretation of individual coefficients in models including both volume terms should consider collinearity and the reduced number of events in time-to-event analyses.

|  | **Multivariable logistic regression (binary endpoint: progression yes/no)** | **Multivariable Cox regression (time-to-progression; censoring at last radiological follow-up)** | **p-value** |
| --- | --- | --- | --- |
| **Dataset / endpoint** | STR cohort (STR_noProgression vs STR_Progression)  Endpoint: progression | STR cohort (STR_noProgression vs STR_Progression)  Endpoint: time-to-progression | — |
| **Model strategy** | Forced-entry of covariates | Forced-entry of covariates | — |
| **Sample size** | Logit-1: n=110  Logit-2: n=109  (both volumes available) | Cox-1/2/3: n=85, events=24 | — |
| **Preoperative tumor volume [cm³]** | Logit-1: OR 1.090 per 1 cm³  95% CI 1.030–1.153  Logit-2: OR 1.139 per 1 cm³  95% CI 1.047–1.239 | Cox-1 (univariable): HR 1.042 per 1 cm³  95% CI 1.014–1.070  Cox-2 (adjusted for postop residual): HR 1.067 per 1 cm³  95% CI 0.982–1.160 | Logit-1: p=0.0027  Logit-2: p=0.0025  Cox-1: p=0.00279  Cox-2: p=0.126 |
| **Postoperative residual volume [cm³]** | Logit-2: OR 0.916 per 1 cm³  95% CI 0.817–1.028 | Cox-2: HR 0.964 per 1 cm³  95% CI 0.854–1.088 | Logit-2: p=0.138  Cox-2: p=0.550 |
| **Surgical approach**(endoscopic vs microsurgical) | Logit-1: OR 2.622  95% CI 0.744–9.241  Logit-2: OR 2.508  95% CI 0.705–8.921 | Cox-3 (added approach): HR 0.987  95% CI 0.342–2.844 | Logit-1: p=0.134 Logit-2: p=0.155 Cox-3: p=0.980 |
| **Model performance / fit** | Logit-1: AUC 0.758  McFadden pseudo-R² 0.125  Hosmer–Lemeshow p=0.856  Logit-2: — | Cox-2: Concordance (Harrell’s C) ~0.725  Cox-3: — | — |
| **Collinearity** | Pre- and postoperative volumes strongly correlated (Pearson r=0.834) VIF (preop/postop volumes) ~3.3 (Logit-2) | VIF (preop/postop volumes) ~5.6 (Cox-2/3) | — |

**Supplementary Table S5.** Baseline characteristics and volumetry in patients with initial GTR. P-values were computed using Mann–Whitney U tests for continuous variables and Fisher’s exact test (binary categorizations) or chi-square tests (where appropriate) for categorical variables.

|  | **Subgroup GTR_noRecurrence (n=62)** | **Subgroup GTR_Recurrence**  **(n=32)** | **p-value** |
| --- | --- | --- | --- |
| **Age [years]** | Mean: 57.13  Median: 61.50  Range: 25 – 85  n=62 | Mean: 51.6  Median: 50.5  Range: 24 – 81  n=32 | p=0.071 |
| **Sex [absolute M/F (%)]** | 40 (64.5%)/22 (35.5%)  n=62 | 17 (53.1%)/15 (46.9%)  n=32 | p=0.373 |
| **Tumor Volume preop [cm3]** | Mean: 5.54  Median: 4.35  Range: 0.60 – 37.20  n=59 | Mean: 10.45  Median: 7.93  Range: 1.29 – 44.30  n=62 | p=0.022 |
| **Time to last radiological follow-up/event [months]** | Mean: 35.9  Median: 15.0  Range: 8-184  n=62 | Mean: 46  Median: 28  Range: 15– 92  n=32 | p=0.018 |
| **Knosp Classification [absolute (%)]** | 0: 1 (1.6 %)  1: 21 (33.9 %)  2: 17 (27.4 %)  3: 23 (37.1 %)  4: 0 (0 %)  n=62 | 0: 0 (0.0 %)  1: 3 (9.4 %)  2: 12 (37.5 %)  3: 13 (40.6 %)  4: 4 (12.5 %)  n=32 | p=0.073 |
| **Hardy Classification [absolute (%)]** | I: 13 (21.0 %)  II: 30 (48.3 %)  III: 6 (9.7 %)  IV: 13(21.0 %)  n=62 | I: 0 (0,0 %)  II: 13 (40.6 %  III: 4 (12.5 %)  IV: 15 (46.9 %)  n=32 | p=0.008 |

**Supplementary Table S6.** Multivariable logistic regression models including preoperative tumor volume and postoperative residual volume.

(A) STR cohort only: outcome = progression. n=109 (available-case dataset with both volumes). ORs are per 1 cm³ increase. Logistic regression with intercept.

| **Predictor** | **OR** | **95% CI** | **p-value** |
| --- | --- | --- | --- |
| **Preoperative tumor volume (per 1 cm³)** | 1.144 | 1.052–1.244 | 0.0016 |
| **Postoperative residual volume (per 1 cm³)** | 0.914 | 0.816–1.025 | 0.1245 |

(B) Whole cohort sensitivity analysis: outcome = any unfavorable outcome (GTR_Recurrence or STR_Progression). n=188 (available-case dataset with both volumes).

| **Predictor** | **OR** | **95% CI** | **p-value** |
| --- | --- | --- | --- |
| **Preoperative tumor volume (per 1 cm³)** | 1.107 | 1.038–1.179 | 0.0018 |
| **Postoperative residual volume (per 1 cm³)** | 0.981 | 0.896–1.075 | 0.6852 |

**Supplementary Table S7.** Exploratory multivariable logistic regression including proliferation markers (available-case sensitivity analyses). Odds ratios (OR) are from multivariable logistic regression (forced entry). Continuous predictors are reported per 1 cm³ increase. Proliferation markers were dichotomized as Ki-67 ≥3%, mitotic activity >2 mitoses per 10 HPF, and p53 positivity >10 strongly positive nuclei per 10 HPF. Analyses used available-case data restricted to patients with non-missing values for all predictors in the respective model.

| **Subset / Endpoint** | **n (events)** | **Predictor** | **OR** | **95% CI** | **p-value** |
| --- | --- | --- | --- | --- | --- |
| **STR cohort / outcome = progression** | 39 (11) | Preoperative tumor volume (per 1 cm³) | 1.148 | 1.016–1.298 | 0.0269 |
|  |  | Ki-67 ≥3% | 0.536 | 0.053–5.425 | 0.598 |
| **Whole cohort / outcome = any unfavorable outcome (GTR_Recurrence or STR_Progression)** | 59 (16) | Preoperative tumor volume (per 1 cm³) | 1.181 | 1.057–1.318 | 0.00316 |
|  |  | Ki-67 ≥3% | 0.291 | 0.017–4.870 | 0.391 |
|  |  | p53 positive | 7.417 | 0.353–155.746 | 0.197 |
|  |  | Mitotic activity positive | 0.817 | 0.049–13.749 | 0.888 |
|  |  |  |  |  |  |

## Supplementary Figures

**Supplementary Figure 1.** Kaplan–Meier progression-free survival (PFS) after subtotal resection (STR). Patients without progression were censored at last radiological follow-up. Landmark PFS at 2 and 5 years is indicated.
